# Evaluation of a Wobbling Method Applied to Correcting Defective Pixels of CZT Detectors in SPECT Imaging

**DOI:** 10.3390/s16060772

**Published:** 2016-05-27

**Authors:** Zhaoheng Xie, Suying Li, Kun Yang, Baixuan Xu, Qiushi Ren

**Affiliations:** 1 Department of Biomedical Engineering, Peking University, No. 5, Yiheyuan Road, Beijing 100871, China; xiezhaoheng@163.com (Z.X.); lisuying90@163.com (S.L.); 2 Department of Control Technology and Instrument, Hebei University, No. 180, Wusi East Road, Baoding 071000, China; yangkun9999@hotmail.com; 3 General Hospital of Chinese People’s Liberation Army, No. 28 Fuxing Road, Beijing 100039, China; xbx301@163.com

**Keywords:** semiconductor detector, CZT, defective points, calibration, wobbling method

## Abstract

In this paper, we propose a wobbling method to correct bad pixels in cadmium zinc telluride (CZT) detectors, using information of related images. We build up an automated device that realizes the wobbling correction for small animal Single Photon Emission Computed Tomography (SPECT) imaging. The wobbling correction method is applied to various constellations of defective pixels. The corrected images are compared with the results of conventional interpolation method, and the correction effectiveness is evaluated quantitatively using the factor of peak signal-to-noise ratio (PSNR) and structural similarity (SSIM). In summary, the proposed wobbling method, equipped with the automatic mechanical system, provides a better image quality for correcting defective pixels, which could be used for all pixelated detectors for molecular imaging.

## 1. Introduction

Radionuclide imaging has become one of the most advanced molecular imaging techniques to monitor physiological functions [1,2]. However, Single Photon Emission Computed Tomography (SPECT) images tend to be “noisy” because of the low amount of radiotracer per volume of interest (VOI) and the effect of Compton scattering in tissue and collimators. Over the past decades, researchers have been dedicated to developing gamma-cameras with improved spatial resolution and energy resolution. Semiconductor nuclear radiation detectors, especially cadmium zinc telluride (Cd_1−x_Zn_x_Te, CZT) materials, have been considered as alternatives to scintillator detectors [3,4,5] because of their good stopping power and low dark current [4,6,7]. Notably, the major advantage of semiconductor over traditional scintillator detectors is that they can directly convert the deposited photon energy into measurable signals, which could improve energy resolution and detection efficiency [4,5,7,8]. Hence, CZT detectors are regarded to be the most promising option for SPECT imaging.

**Figure 1:**
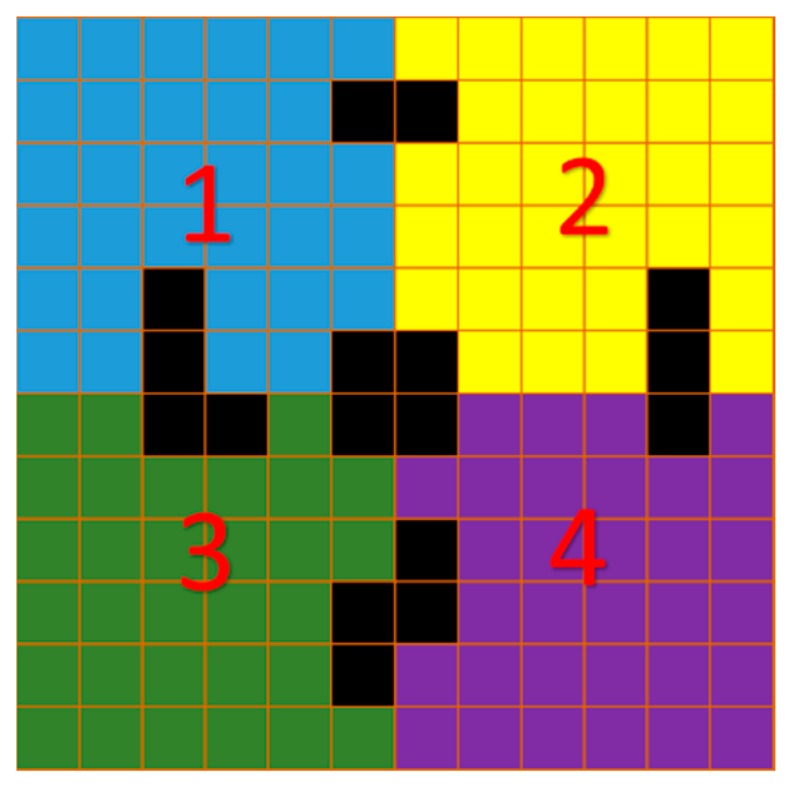
Example of different defective pixel patterns in multi-module pixelated detector; 1, 2, 3, 4 means four adjacent detector modules, black squares represent cluster of defective pixels.

The CZT detector consists of a semiconducting crystal that is bump-bonded to a large area ASIC and packaged with a high performance data acquisition system. Recently, great progress has been made in electronics [9,10,11,12] and crystal growing [13,14]. However, there are still some defective pixels, showing degraded features, such as split or broadening spectral peaks and extraordinarily low or high response, especially for pixels with smaller sizes or at the edge of module. Though the defective pixels only occupy a small proportion of each module, separate defects of each module may cluster to form continuous defective regions if several modules are arranged into a large detector, as shown in Figure 1. The continuous defective region in detector occurs in projections and introduces ring artefacts [15] after reconstruction, which degrades image quality and may lead to misinterpretation, e.g., misdiagnosis or overdiagnosis [16].

Interpolation is a common correction method for defective pixels, which may work well for separate or individual bad pixels in homogeneous regions. For bad pixel cluster regions, however, simple interpolation often leads to inaccurate estimations. The determination of interpolation direction also poses more complexity since there are many variations for the constellation of defective pixels. Moreover, the advantage of interpolation method vanishes when the pixel size is small [17]. A proposed alternative plan is using sinogram-processing to eliminate the ring artifacts. Nonetheless, the 2D-wavelet-analysis [18] and polyphase decomposition in sinogram process [19] may introduce some noise or artifacts, and it has higher numerical complexity.

In this paper, an advanced wobbling method is proposed to correct the defective pixels. In order to accomplish the correction method and demonstrate its effects, we built a CZT SPECT system equipped with a simple mechanical device and conduct phantom experiment. The correction method is applied to various constellations of defective pixels. The correction effectiveness is evaluated quantitatively and is compared with conventional interpolation method. The results proved that the proposed wobbling method provides improvement in image quality of pixelated semiconductor detector, especially for the small object imaging with pinhole collimator.

## 2. Materials and Methods

### 2.1. Wobbling Correction Method

The wobbling method mainly includes following four steps:

#### 2.1.1. 1st Step: Uniformity Correction and Wobbling Path Planning

The uniformity corrections are conducted by flood phantom first. From the flood image, some continuous bad pixels, which would affect image quality and cause ring artifacts after reconstruction, have been localized. Then all of defective pixels are recognized and the wobbling path is defined.

#### 2.1.2. 2nd Step: Acquisition of the Wobbling Images

**Figure 2:**
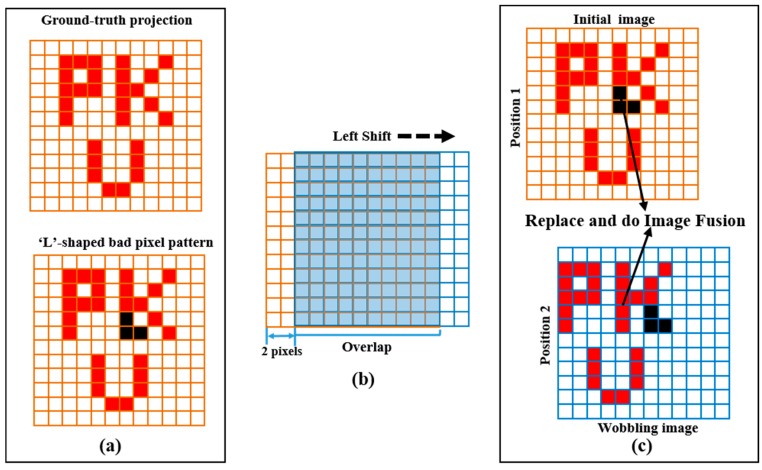
Description of the method for the wobbling technique. (**a**) Example of image without defectives and “L”-shaped bad-pixel pattern in detector; (**b**) The sketch of wobbling acquisition with Left shift; (**c**) Two images of different positions for the same object (Black points represent defective pixels in the fixed position of detector).

The basis of the wobbling correction method is the acquisition of wobbling images. Typically, it acquires different observations of the same object, *i.e.*, images with shifts of several pixel dimensions. Figure 2 shows an example of wobbling acquisition for the method. It is assumed that the projection on the detector is a “PKU”-shaped image and an “L”-shaped bad pixel pattern exists on CZT detector, as shown in Figure 2a. The detector performs a scan at Position 1 and subsequently acquires a wobbling image after a left shift of two pixel dimension in the horizontal direction (Figure 2b). In this way, we get the related images (“twin images”, as shown in Figure 2c). Each of the wobbling images contributes useful information to the final corrected image and its correlation will be illustrated in the following 3rd Step.

The wobbling acquisition can perform vertical and horizontal movement according to the defective-pixel patterns. In this paper, we mainly considered two-pixel shifts to correct defective pixels, so that there are 72.5% overlap between the two related images.

#### 2.1.3. 3rd Step: Registration of Images Obtained from the Wobbling Method

After wobbling acquisition, each projection has two samples (images A and B), which respectively represent the initial and wobbling images, regarding the same imaging object. $f_{A}\left(x_{A},y_{A}\right)$ and $f_{B}\left(x_{B},y_{B}\right)$ are the number of emitted photons which are detected by each pixel. (x,y) means pixel index. The correlation between *f_A_* and *f_B_* can be expressed as a general rigid-body transformation that includes a combination of rotation and translation:

(1) $$f_{B}(x_{B},y_{B})=R_{AB}\cdot f_{A}(x_{A},y_{A})+T_{AB}$$

where $R_{AB}$ and $T_{AB}$ is the translate transformation and rotate transformation between $f_{A}\left(x_{A},y_{A}\right)$ and $f_{B}\left(x_{B},y_{B}\right)$. A rigid-body transformation is applicable provided the pixel size of the CZT detector is negligible compared with the accuracy of the linear translation stage.

#### 2.1.4. 4th Step: Replace the Defective Pixels and Apply an Image Fusion Algorithm

**Figure 3:**
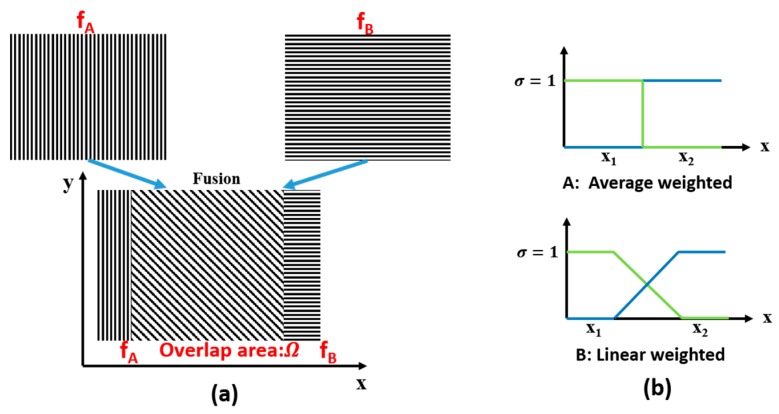
Image fusion algorithms and examples of different weighted function. (**a**) The overlapped regions are indicated by the slant dashed line. The two images on the left and right are to be stitched; (**b**) represents distinctive average features in overlapped region Ω. R and L are the region viewed exclusively in image $f_{A}$ and $f_{B}$ (L∩R=L∩Ω=R∩Ω=∅).

After establishing the point-to-point correspondence of $f_{A}$ and $f_{B}$, we replace defective pixels using their counterparts and apply an image fusion algorithm to the two related images. The basic case of two roughly aligned images $f_{A}$ and $f_{B}$ with the overlapped area Ω is shown in Figure 3. In this paper, we mainly investigate the following two approaches to produce the mosaic image *I*_Ω_:

(1) Simple averaging function(Fusion method A):
  (2) $$I_{\Omega }=\frac{1}{2}f_{A}(x_{A},y_{A})+\frac{1}{2}f_{B}(x_{B},y_{B})$$
(2) Distance weighted function(Fusion method B):
  (3) $$I_{\Omega }=\sigma f_{A}(x_{A},y_{A})+(1-\sigma )f_{B}(x_{B},y_{B})$$
  where σ(0 ≤ σ ≤ 1) is the transition factor, as shown in Figure 3, based on the distance of the current pixel coordinate from its own boundary.

The trade-off relationship between image quality and acquisition time is also considered here. Compared with the common acquisition mode, the acquisition time of each scan in the wobbling method is halved for two positions. In this way, the wobbling method can acquire sufficient counts without extending the data acquisition time. In this study, we are mainly interested in how to correct fixed defective pixels and edge-effects in CZT detectors, other defects such as geometric deformation, noise corruption and subsequent reconstruction algorithms, are not considered here.

### 2.2. System Description

**Figure 4:**
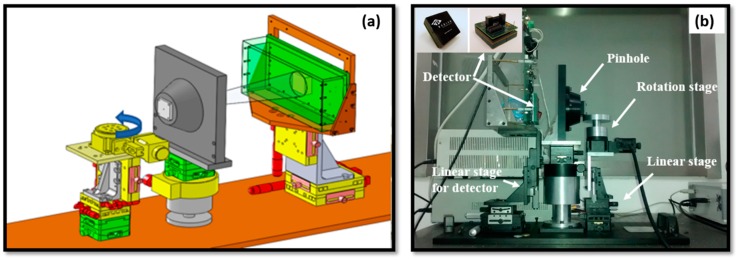
(**a**) Schematic diagram of the pinhole SPECT system design, which consists of rotary stage, collimator and CZT detector; (**b**) Close-up of the pinhole SPECT system.

The pinhole SPECT system is developed based on a CZT-detector and aiming for small animal imaging. The system, as shown in Figure 4, contains the detector, pinhole collimator and rotation stage where imaging objects are placed. The single pinhole collimator is made of tungsten alloy (ρ = 18.5 g/cm^3^), with 0.8 mm aperture diameter, 1.38 mm channel height, and 60° opening angle. A linear translation stage is installed underneath the detector that provides support for the detector and the drive for wobble motion. It allows linear movement along the X, Y axes within a 50 mm range and its precision can reach 20 μm. The stage contains a grating ruler, which gives the feedback of motion position and guarantees the precision. With the translation stage, the wobbling range of detector can be adjusted from 0.25 to 50 mm in steps of 0.25 mm. The movement of linear stage is controlled by dedicated C++ codes, which are integrated into the acquisition software. After the first position the detector performs a scan and is subsequently wobbled for the following acquisition. Each scan is saved for post-processing according to the described wobbling method in Figure 2. A standard M1522 CZT detector module (Figure 4), has an active region of 40 × 40 mm^2^ and 5 mm thick with Au contact (Redlen Technologies Inc., Saanichton, BC , Canada) was used in this work. The module is organized in a 16 × 16 array with a 2.46 mm pixel pitch and its acquisition software provides pixel position and energy value in a binary format. Energy resolution of this module is typical about 6.5% (Co-57 source).

### 2.3. Phantom Experiment

#### 2.3.1. Flood Image Experiment

Flood images are obtained to check the performance of the CZT detector. We arranged four CZT modules into a 2 × 2 array, resulting in an 80 × 80 mm^2^ detection area. It was exposed to a Tc-99m flood source with 2.22 mCi for 3600 s. The flood images are obtained with the 15% energy window (140 keV). The uniformity and energy spectrum of individual pixel as well as whole entire detector have been analyzed.

#### 2.3.2. Line Source Phantom Experiment

**Figure 5:**
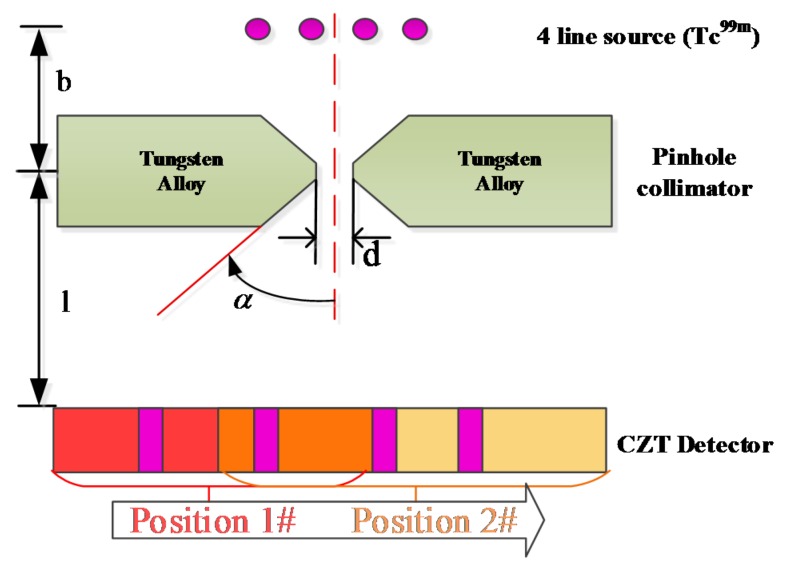
The geometry of keel-edge pinhole aperture and four linear sources phantom, where *b* is the perpendicular distance between source and the focal point (pinhole center), *l* is focal length, *d* is the physical diameter of pinhole, α is half of the pinhole opening angle.

To evaluate the performance of the proposed correction method, line source phantom experiment is designed as shown in Figure 5.

Four line sources (2 mm inner diameter) are placed off-axis, symmetrically on the two sides of axis of rotation (AOR) which is 60 mm away from the aperture (120 mm from the detector surface). The center distance between each source is 4 mm. This geometric configuration approximately results in 2× magnification. Each line source is filled with 2.6 mCi/mL Tc-99m labelled medronate (MDP). After data acquisition, the correction and evaluation processes are performed by a Matlab-based program.

### 2.4. Image Quality Evaluation

Aiming at evaluating the effect of our wobbling correction method, we conducted phantom experiments and compared the results with a conventional correction method. Firstly, line sources are scanned statically as reference images. On the reference images, we deliberately defined some pixels as bad pixels and set the counts as zero, resulting in the image waiting to be corrected (identified as “bad image” in the following). Correction methods are applied to various bad-pixel patterns such as: (1) two bad pixels (vertical); (2) two bad pixels (horizontal); (3) three bad pixels; (4) four bad pixels. Here, we mainly investigate the effect of the wobbling method in the correction of bad pixels that appear in the region of interest (ROI) and appear in the background. We defined the effectiveness of a correction method as the similarity between the corrected image (C) and reference image (R). The similarity is assessed using peak signal-to-noise ratio (PSNR) and structural similarity (SSIM), which are the widely used full-reference quality metrics [20,21]. Given the reference image R and the corrected image C, both of size M × N, the PSNR between C and R is defined as:

(4) $$\text{PSNR}(C,R)=10{\text{log}}_{10}(255^{2}/\text{MSE}(C,R))$$

where:

(5) $$\text{MSE}(C,R)=\frac{1}{\text{MN}}\sum_{i=1}^{M}\sum_{j=1}^{N}\left(C_{ij}-R_{ij}\right)$$

When MSE, which is short for mean squared error, approaches zero, the PSNR value approaches infinity, thus a higher PSNR value provides a higher image quality.

The SSIM is a well-known quality metric used to assess the similarity between two image, considering a combination of three factors that loss of correlation, luminance distortion and contrast distortion. The SSIM is defined as:

(6) $$\text{SSIM}(C,R)={\left[l(C,R)\right]}^{\alpha }\cdot {\left[c(C,R)\right]}^{\beta }\cdot {\left[s(C,R)\right]}^{\gamma }$$

where l(C, R) is the luminance comparison function, c(C, R) is contrast comparison function, and s(C, R) is structure comparison function and they are calculated as following equations:

(7) $$l(C,R)=\frac{2\mu_{C}\mu_{R}+c1}{{\mu_{C}}^{2}+{\mu_{R}}^{2}+c1},c(C,R)=\frac{2\sigma_{C}\sigma_{R}+c2}{{\sigma_{C}}^{2}+{\sigma_{C}}^{2}+c2},s(C,R)=\frac{\sigma_{CR}+c3}{\sigma_{C}\sigma_{R}+c3}$$

Moreover, α, β, γ in Equation (7) are parameters used to adjust the relative importance of the three factors and the positive values constants c1, c2, c3 are used to avoid a null denominator. In order to simplify the expression, we set α = β = γ = 1 and c1 = c2 = 2c3 in this paper. We use PSNR to assess the correction effect of pixels that appeared in ROI and use SSIM in terms of bad pixels appearing in the background. Here, we execute the wobbling correction method using averaging and distance-weighting fusion models (mentioned as “Method A” and “Method B”) and compare the results with a conventional interpolation correction method.

## 3. Results

### 3.1. Flood Image Result

**Figure 6:**
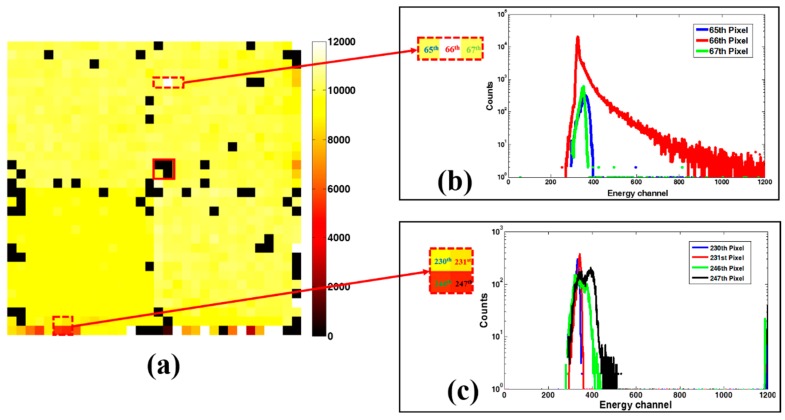
Spectra and counts are measured by flood phantom from a 2 × 2 modules of detector. (**a**) The Tc-99m flood image, the black spot (no counts) in the detector area (solid box) is caused by a cluster defect region. The white and brown spots (dashed box) in the image can be attributed to irregular response pixels; (**b**) Extreme high response pixels compared with adjacent normal pixels; (**c**) Pixels which show split or broadening spectral peak at the edge of detector.

A typical image is shown in Figure 6. In the following pairs of spectra, we describe the different pixels’ behavior. The black spots in Figure 6a represent dead pixels which have resulted in a large reduction of system sensitivity. Otherwise, pixels with split spectrum (247th pixel), broadening peaks (246th pixel) or non-standard response (65th pixel) are compared with “normal” pixels in Figure 6b,c. We found the unusual response pixels tend to bunch together, especially at the edge of the module. This may be attributed to the effects of local crystal structure variations. Overall, defective pixels represent less than 10% of all the pixels.

### 3.2. Line Source Image Result

**Figure 7:**
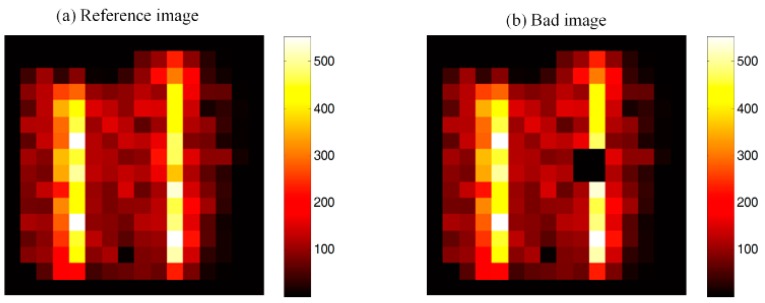

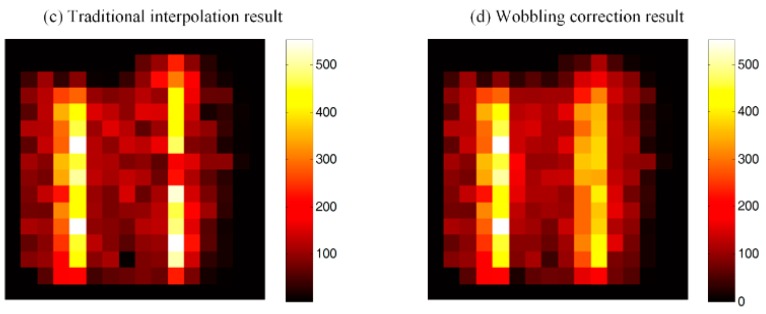
Results of the acquired line source phantom and correction results comparison. (**a**) Reference image: static imaging of line sources; (**b**) Bad image: image with 4 continuous bad pixels; (**c**) Conventional interpolation result: image using interpolation method; (**d**) Wobbling correction result: image using wobbling correction method.

**Figure 8:**
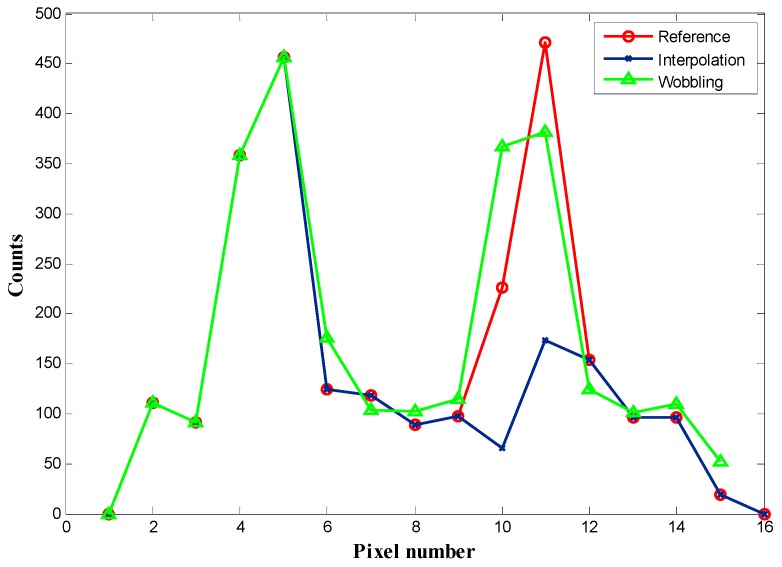
The pixel counts across row 8th of the corrected images. The blue line indicates the pixel counts after conventional interpolation and the green line indicates the counts after the proposed wobbling method is applied. The red line is the counts of the reference image.

Figure 7 displays an example of the application of the wobbling correction method to four continuous bad pixels on the detector. A line source image is acquired statically as reference image (Figure 7a). Then we define four continuous bad pixels in ROI (8th and 9th row, 10th and 11th column) as Figure 7b. Figure 7c,d is the corrected image using a conventional interpolation method and the proposed wobbling correction method, respectively. Figure 8 compares line profiles of the pixel counts across the 8th row (the bad pixel location) after application of the different correction methods. The interpolation results in an obvious decrease of counts and information loss around the bad pixel region. Contrarily, the wobbling correction result shows similar counts as the reference image and recovers the missing information more effectively. The phantom results are valid examples to highlight the enhancement of the wobbling correction method.

## 4. Discussion

**Figure 9:**
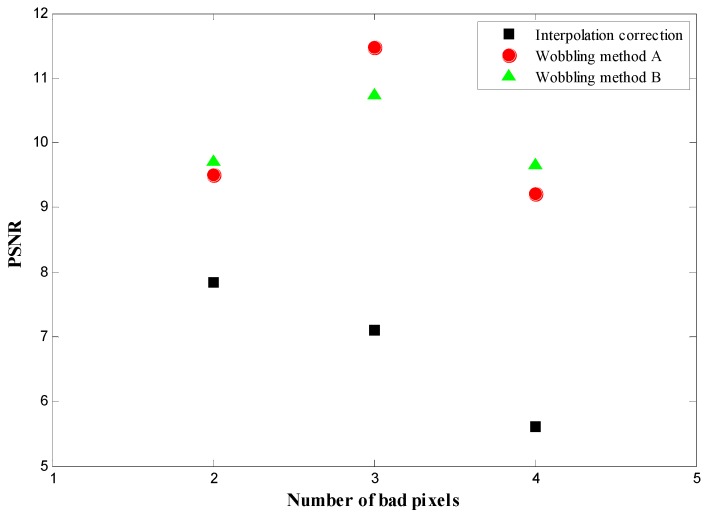
PSNR of different correction methods for various bad-pixel numbers.

**Figure 10:**
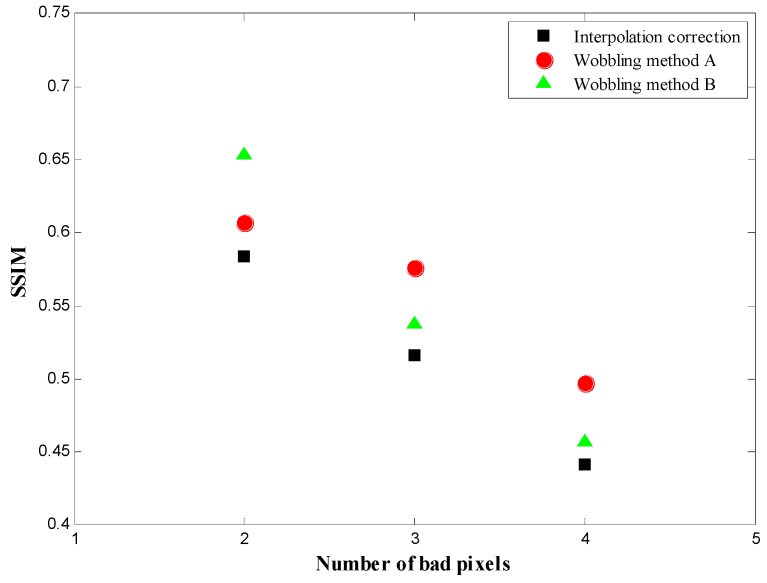
SSIM of different correction methods for various bad-pixel numbers.

According to the evaluation factor defined in Section 2.4, the PSNR and SSIM are calculated for various bad pixel conditions with different pixel numbers and arrangements. The PSNR results of bad pixels appearing in the ROI are listed in Table 1. The SSIM results, assessing the correction effect for background bad pixels, are listed in Table 2. The improvements of the wobbling methods A and B compared with the conventional interpolation method are also marked in Table 1 and Table 2. In order to demonstrate the improvement of the wobbling correction method explicitly, the PSNR and SSIM values of different correction methods for various bad-pixel numbers are plotted in Figure 9 and Figure 10, respectively.

The proposed wobbling correction method results in higher PSNR and SSIM, in particular when there are more than two continuous bad pixels. For the condition where bad pixels appear in the ROI, the improvement of the wobbling method results is around 10%~20% when the number of bad pixels is less than three. As the number of continuous bad pixels gets larger, he PSNR of the proposed wobbling method is significantly higher. For three continuous bad pixels, the PSNR of the wobbling method A and B is 1.6 and 1.5 times higher than the interpolation result. According to Table 2 of SSIM results, the overall improvement of the wobbling method is 10% for correction of background bad pixels, compared with conventional interpolation. As can be seen in Figure 8 and Figure 9, defects up to 4 bad pixels can be corrected almost flawlessly when the wobbling correction is used. Conventional interpolation may already be inadequate for bad-pixel numbers up to four. This is because the conventional interpolation method is based on using the neighboring pixels to estimate the bad pixels’ counts. When the defects cluster to form large regions of corrected pixels, the neighboring information is not sufficient for the correction, whereas the proposed method acquires two images of related wobbling positions, which can provide a comprehensive reference for correction, so the wobbling method is still effective when there are large defective regions. Moreover, the proper fusion model in the wobbling method can make effective use of the two wobbling images, producing desirable correction results.

## 5. Conclusions

In this paper, we propose a novel method for correcting continuous bad pixels. Within the same acquisition time, images of two wobbling positions provide a reference for reasonable correction. We conduct corrections for various bad pixel conditions and use PSNR and SSIM estimators to evaluate the correction results. In the phantom experiment, the conventional interpolation method is used for comparison. The results show that wobbling method can correct continuous bad pixels effectively, no matter whether they appear in the ROI or background, whereas, a conventional interpolation method cannot effectively recover the original information, especially when there are more than three continuous bad pixels. In our future study, a proper image fusion algorithm of the wobbling images can be further investigated, resulting in a more effective correction. The wobbling method is conceptually simple, computationally efficient, and easy to use. Furthermore, this correction technique is potentially applicable to the standard pixelated detector, such as Si, GaAs and CdTe, with a proper motorized system.

## Figures and Tables

**Table 1:** PSNR of correction for bad pixels appearing in the ROI.

Number of Bad Pixels	Shape	PSNR (Improvement %)		
		Conventional Interpolation	Method A	Method B
**2**	Vertical	6.3953	6.8457 (+7%)	7.3356 (+14.7)
**2**	Horizontal	7.8373	9.5113 (+21%)	9.7114 (+23.9%)
**3**	“L”-shaped	7.1087	11.4718 (+61.4%)	10.745 (+51.2%)
**4**	Square	5.6144	9.2081 (+64%)	9.6518 (+71.9%)

**Table 2:** SSIM of correction for background bad pixels.

Number of Bad Pixels	SSIM (Improvement %)		
	Conventional Interpolation	Method A	Method B
2	0.584	0.607 (+3.9%)	0.6532 (+11.8%)
3	0.5166	0.5767 (+11.6%)	0.5374 (+4%)
4	0.4419	0.497 (+12.5%)	0.4574 (+3.5%)

## Abbreviations

The following abbreviations are used in this manuscript:

CZT	Cadmium zinc telluride
SPECT	Single Photon Emission Computed Tomography
VOI	Volume of interest
SNR	Signal-to-noise
PSNR	Peak signal-to-noise ratio
MSE	Mean squared error
SSIM	Structural similarity
